# Control of social status by sex steroids: insights from teleost fishes

**DOI:** 10.12688/molpsychol.17571.1

**Published:** 2023-09-28

**Authors:** Kathleen M. Munley, Beau A. Alward

**Affiliations:** 1 Psychology, University of Houston, Houston, Texas, 77204, USA; 2 Biology and Biochemistry, University of Houston, Houston, Texas, 77004, USA

**Keywords:** androgens, aggression, estrogens, fish, gene editing, reproduction, social rank, steroids

## Abstract

Many animals live in highly social environments, in which individuals must behave in a way that enables them to survive and live harmoniously among conspecifics. Dominance hierarchies are typical among social species and are essential for determining and preserving stability within social groups. Although there is considerable evidence that sex steroid hormones regulate behaviors associated with dominance, such as aggression and mating, fewer studies have examined the role of these hormones in controlling social status, especially in species that exhibit social hierarchies. Furthermore, despite this research, we know remarkably little about the precise neural and molecular mechanisms through which sex steroids modulate traits associated with social rank. Here, we review the neuroendocrine regulation of social status by sex steroids in teleost fishes, the largest and most diverse vertebrate group that shows extensive variation in reproductive systems and social structures between species. First, we describe the function of sex steroids and novel steroid-related genes that teleost fishes possess due to a lineage-specific whole-genome duplication event. Then, we discuss correlational, pharmacological, and molecular genetic studies on the control of social status by sex steroids in teleost fishes, including recent studies that have implemented gene editing technologies, such as CRISPR/Cas9. Finally, we argue that gene editing approaches in teleost studies, within both integrative and comparative frameworks, will be vital for elucidating the role of sex steroids in controlling social rank and characterizing their neural and molecular mechanisms of action. Collectively, ongoing and future research in these species will provide novel insight into the evolution of the regulation of social status by sex steroids and other neuroendocrine substrates across vertebrates.

## 1. Introduction

Hierarchies are a common feature among social organisms^1–3^. Rank along a social hierarchy impacts which members of a group have access to shelter and mates and other resources, such as food. While social hierarchies can vary considerably in size and composition, they generally consist of higher-ranking dominant (DOM) individuals, which tend to have greater access to resources such as food and shelter and more opportunities for mating compared to lower-ranking subordinate (SUB) individuals^2,4,5^. Rank within a hierarchy is determined by frequent social interactions among individuals and is associated with distinct physiological and behavioral traits^6,7^. These hierarchical structures, however, are dynamic and can be altered due to naturally occurring changes in the social environment (e.g., predation, death). Thus, for many species individuals may have the chance to rise in social rank (i.e., social ascent) or descend in social rank (i.e., social descent). In addition to enhancing social stability, social hierarchies can have both positive and negative impacts on mental and physical health^2^. The influence of social and environmental cues on social rank has been studied extensively for decades in a variety of vertebrate species, including mice^8,9^, rats^10–14^, fishes^15^, lizards^16–18^, and primates^2^. Variation in numerous species-specific traits modulated by the social environment influence an individual’s social rank, such as body size, social behaviors, and territory ownership. Well-known physiological correlates of social rank include a positive association between sex steroid hormone levels (e.g., androgens and estrogens), gonad mass, and territorial and mating behaviors.

Despite what is known about the behavioral and physiological traits linked to social rank, the molecular and neural mechanisms governing social status are unclear. The mechanisms controlling DOM-typical social behaviors (e.g., aggression, mating), however, have been investigated quite extensively. These studies have shown that androgens and estrogens control aggression and mating behaviors across vertebrate species^19,20^. Moreover, there is considerable evidence that steroid hormone levels differ with social status and that correlations between steroids and behaviors associated with social rank are stronger during periods of social instability (e.g., establishment of dominance hierarchies or territories, competition)^21–24^. Fewer studies have characterized the molecular and neural mechanisms that regulate social status specifically, although recent work has begun to shed light on some of the processes involved^25–27^. Given the strong link between sex steroid hormones and social rank across vertebrate species, investigating how sex steroid signaling systems control social status is warranted. Additionally, sex steroid receptors and their ligands have relatively high homology among vertebrates^28,29^ and follow conserved expression patterns in the brain^19,30,31^. Therefore, studying the regulation of social status by sex steroid hormones within a comparative framework may lead to important discoveries on the fundamental mechanisms underlying key aspects of social status across species. One of the most diverse groups of species for which comparative work may be especially fruitful in this regard are teleost fishes. In this review, we highlight research that has examined the relationships between sex steroid hormone signaling and social status in teleosts. Given the diversity of life histories, species-specific variations in social hierarchies, and the availability of state-of-the-art genetic tools among these species, teleost fishes are an excellent group for enhancing our understanding of the control of social status by sex steroid hormones. We begin our review by briefly describing the general functions of steroid hormones and the unique steroid signaling genes that teleosts due to a lineage-specific whole-genome duplication. Then, we discuss correlational, pharmacological, and genetic studies that provide insights into the control of social status by sex steroid hormones in teleost fishes. We assert that the use of teleost fishes as model organisms, along with the integration of gene editing methodologies, will be critical for elucidating the functions of sex steroid hormones in governing social status across species.

## 2. A primer on steroid hormones in teleost fishes

### 2.1. Brief background on steroid mechanisms of action

Steroid hormones are a group of lipophilic molecules with a cholesterol backbone that can act as transcription factors or secondary messengers by binding to membranous, cytoplasmic, or nuclear receptors^32^. Steroidogenesis occurs in peripheral tissues and specific brain regions through the actions of enzymes that synthesize steroids *de novo* from cholesterol or from prohormones in circulation^33^. Prohormones are often found at high levels in the blood and have lower affinity to receptors compared to their more active metabolites^34,35^. Thus, the conversion of prohormones to more potent steroids within target tissues is critical to modulating physiological processes^36^. For example, the androgen testosterone can be converted to both androgenic and estrogenic metabolites, including the more potent steorids such as 5α-dihydrotestosterone via the enzyme 5α-reductase; the androgen 11-ketotesteosterone (11-KT) via the enzymes 11-hydrozysteroid dehydrogenase types 1 and 2, which is the main androgen in teleost fishes^37^; and the estrogenic metabolite 17β-estradiol (E_2_) via the enzyme aromatase^36,37^.

Steroid receptors modulate a multitude of processes, such as cellular signaling, body temperature and other homeostatic functions, sexual differentiation, and social behaviors. These processes are controlled through steroid receptors acting through one or two mechanisms: 1) a “genomic” mechanism in which steroids bind to cytoplasmic or nuclear receptors, and 2) a “non-genomic” mechanism where steroids activate a second messenger cascade or modify membrane-located ionic receptors. For example, testosterone can exhibit genomic mechanisms of action by binding to the androgen receptor, a process that results in dimerization and the formation an androgen-receptor complex that then binds to a nuclear receptor to modulate gene expression. Conversely, non-genomic mechanisms of action may lead to indirect changes in gene expression depending on the secondary messenger system or specific ion channels that are activated. Collectively, there are five major classes of steroid receptors, including androgen receptors (ARs), estrogen receptors (ERs), glucocorticoid receptors (GRs), mineralocorticoid receptors (MRs), and progesterone receptors (PRs). Like steroidogenic enzymes, steroid receptors are expressed in the periphery and in specific regions of the brain.

### 2.2. Teleosts possess novel steroid receptors resulting from a teleost-specific whole-genome duplication

About 350 million years ago, the common ancestor of all teleost fishes underwent a whole-genome duplication event^38,39^. This teleost-specific whole-genome duplication (TS-WGD) led to the replication of many important genes. After a whole-genome duplication event, a redundant gene most commonly experiences relaxed selective pressure, which leads to the buildup of deleterious mutations and culminates in nonfunctionalization^40^. Alternatively, positive selection may lead to neofunctionalization, in which a gene paralog accumulates mutates that confer novel functions^40,41^. A third outcome is subfunctionalization, whereby ancestral functions are partitioned between the two gene paralogs^41,42^.

The TS-WGD led to novel duplicates (also referred to as “paralogs”) encoding steroidogenic enzymes and steroid receptors, which may have a profound impact on the diversity of reproductive strategies and social structures among teleost fishes^43–46^. While all other vertebrates possess one AR encoded by a single gene, Most teleost fishes express two distinct ARs (ARα and ARβ), which are encoded by two different genes (*ar1* and *ar2*)^20^ and extra copies of ERs. Indeed, like other vertebrates, teleosts possess ERα and ERβ, which are encoded by the genes *esr1* and *esr2*, respectively; however, many teleosts possess an additional copy of ERβ. Therefore, teleosts have two ERβs (ERβ1 and ERβ2) that encoded by *esr2a* and *esr2b*. Numerous teleost species also possess an extra GR: GRα and GRβ, which are encoded by the *nr3c1a* and *nr3c1b* genes, respectively. Like other vertebrates, teleost fishes only have one MR. Most teleost fishes have one PR, but goldfish have two encoded by two genes. As observed for steroid receptors, teleost fishes also possess duplicate genes of many steroidogenic enzymes. For example, in teleosts, the two aromatase genes, *cyp19a1* and *cyp19a1a*, are highly conserved and contrast with all other vertebrates, which only possess one aromatase gene. Across teleost species, the functions of these steroid systems have been studied using both pharmacology and genetic manipulation. To date, the majority of research assessing the role of steroid hormones in regulating social behaviors and social status have been correlational and pharmacological in nature; thus, genetic manipulations to understand steroid hormone functions in behavior are needed.

## 3. Neuroendocrine control of social status by sex steroids in teleosts

Many teleost fishes live in social groups, in which social stability is maintained via hierarchies in one or both sexes^47,48^. In this section, we discuss themes and variations that have been revealed about the role of steroid hormones in modulating behaviors associated with social status through studies of teleost fishes, specifically by highlighting correlational, pharmacological, and genetic studies of the neuroendocrine regulation of social status by steroids. We focus our review on sex steroids (androgens, estrogens, and progestins) as modulators of DOM-typical behaviors (e.g., aggression, mating), which have been major emphases of studies investigating how steroids control social status in teleosts for the past several decades (for more information on the regulation of social status by glucocorticoids in teleost fishes and other vertebrates, see these reviews:^49–52^).

### 3.1. Associations between sex steroids and social rank

T and 11-KT have been extensively studied for their roles in regulating aggression and mating in teleosts, including species that exhibit social hierarchies (Table 1)^23,24^. Numerous studies have shown that DOM teleost males have higher circulating androgen levels than SUB males, including African cichlids [Burton’s mouthbrooder (*Astatotilapia burtoni*)^53–57^; Mozambique tilapia (*Oreochromis mossambicus*)^58^; Nile tilapia (*Oreochromis niloticus*)^59^; daffodil cichlid (*Neolamprologus pulcher*)^60–62^; Dimerus cichlid (*Cichlasoma dimerus*)^63,64^; Nyerere’s Victoria cichlid (*Pundamilia nyererei*):^65^], salmonids [rainbow trout (*Oncorhynchus mykiss*)^66,67^; brown trout (*Salmo trutta*)^66^; Arctic char (*Salvelinus alpinus*)^68^], reef fish [stoplight parrotfish (*Sparisoma viride*)^69^; New Zealand demoiselle (*Chromis dispilus*)^70^; ocellated wrasse (*Symphodus ocellatus*)^71^], Japanese medaka (*Oryzias latipes*)^72^, and zebrafish (*Danio rerio*)^73^. Moreover, levels of DOM-typical behaviors, such as male-male aggression and reproductive behaviors, are positively associated with circulating T and/or 11-KT in some of these species (e.g., *A. burtoni*^74,75^; *C. dimerus*^76^). Similar relationships between circulating androgen levels and aggressive and reproductive behaviors have also been documented in some teleost species that do not readily form social hierarchies, including Siamese fighting fish (*Betta splendens*)^77,78^, peacock blennies (*Salaria pavo*)^79,80^, swordtail fish (*Xiphophorus helleri*)^81^, white perch (*Morone americana*)^82^, Gulf toadfish (*Opsanus beta*)^83^, plainfin midshipman (*Porichthys notatus*)^84^, and three-spined stickleback (*Gasterosteus aculeatus*)^85,86^. Collectively, these studies suggest a conserved role of androgens in controlling DOM-typical behaviors, including aggression and mating, in some species of teleost fishes.

In addition, androgens can be influenced by the social environment, even within a given social rank, which can affect the organization and stability of a dominance hierarchy within a population^98–100^. For example, DOM males exhibit an increase in circulating T and/or 11-KT following inter-male agonistic interactions in many teleost species (*A. burtoni*^54^; *N. pulcher*^62,101^; *O. mossambicus*: Oliveira *et al.*, 2009^102,103^; *P. nyererei*^65^; callipterus cichlid (*Lamprologus callipterus*), blunthead cichlid (*Tropheus moorii*), and *Pseudosimochromis curvifrons*^104^; *D. rerio*^105,106^. Moreover, higher baseline levels of circulating androgens in DOM males can reinforce their status by increasing their chances of winning future agonistic interactions, a phenomenon called the winner effect (*O. mossambicus*^102^; *Pundamilia sp*^107^). Prior work also suggests that androgens are altered by social context, including exposure to other social interactions within the population or environmental cues that may proceed subsequent territorial intrusions (i.e., the bystander effect; *O. mossambicus*^106,108^), the presence of conspecifics during an agonistic encounter (i.e., audience effects; *O. mossambicus*^109^; *B. splendens*^77^), and the familiarity with the opponent (i.e., the dear enemy effect; *O. mossambicus*^110^). Together, these studies demonstrate how circulating T and 11-KT are affected not only by social status, but also by the social interactions that individuals encounter in their environment.

Although few studies have examined how estrogens and progestins vary based on social status, there is some support that these hormones are linked with a DOM social rank and its associated behaviors in cichlids^23^. Circulating levels of E_2_ and progestins are higher in DOM males than SUB males in *A. burtoni*^56,111^. Furthermore, E_2_ and progestins are positively correlated with aggressive and reproductive behaviors in DOM male *A. burtoni*^75^. It is important to note, however, that similar relationships between estrogens, progestins, and social status are not exhibited by all cichlids. For example, DOM male *C. dimerus* have lower circulating E_2_ than SUB males, yet DOM males increase plasma E_2_ levels following an aggressive interaction^64,112^. Collectively, these findings suggest that circulating estrogens and progestins are linked with social status in cichlids, including aggression and mating behaviors, but that the direction of these correlations are species-specific. Given the paucity of research on how circulating estrogens and progestins are associated with social status and aggressive and reproductive behaviors, additional studies are necessary to investigate these potential relationships in other teleost species, including those that exhibit and do not exhibit dominance hierarchies.

### 3.2. Pharmacological studies to understand the regulation of social status by sex steroids

In several teleost species, pharmacological manipulations have been used to study the role of sex steroid hormones in regulating aggressive and mating behaviors. Many of these studies use drugs that modify the activity of ER, AR, or PR signaling. A considerable array of work in zebrafish has used synthetic estrogenic analogs to determine their impact on behavior. Other studies have used AR and PR agonists and antagonists to determine the role of these receptor systems on aggressive and mating behaviors. The roles played by sex steroids in modulating aggression, mating, and social status has been studied in-depth in *A. burtoni*, our lab’s study species. In this section, we summarize pharmacological manipulations conducted across teleost species to understand the regulation of social status by sex steroids, with an emphasis on findings in *A. burtoni.*

Zebrafish aggressive and mating behaviors have been studied extensively^113^. Most pharmacological findings on the role of steroid hormones in regulating aggression and mating in this species are from experiments performed within a toxicology framework^88^. For example, the effects of the synthetic estrogen analogue ethinyl estradiol (EE2), a main ingredient in most birth control pills, and Bisphenol A (BPA), an endocrine disrupting chemical (EDC) found in industrial plastics, have been tested on both male and female zebrafish. BPA has been shown to act as an ER agonist, an AR antagonist, and an inhibitor of T synthesis^114^. In male zebrafish, EE2 treatment enhances aggression in a mirror assay and reduces social preference in a social cohesion assay^115^, whereas BPA administration reduces courtship behavior, but increases aggression towards male conspecifics^116^. Similar results have been found in medaka species: E_2_ injections and EE2 treatment significantly reduce mating behavior in male Japanese medaka^97,117^, and EE2 administration decreases reproductive behaviors in mating pairs of brackish medaka (*Oryzias melastigma*)^118^. Similar to findings in medaka and zebrafish, EE2 treatment disrupts reproductive and aggressive behaviors in male fathead minnows (*Pimephales promelas*)^93^. Males given EE2 showed a reduced ability to compete for and clean spawning sites and exhibit a lower frequency of chasing behavior directed towards male conspecifics.

Additional insights into the neuroendocrine regulation of aggression and mating have been gained from research that has altered androgen levels using synthetic compounds. For example, Belanger *et al.* tested the effects of the aromatizable synthetic androgen 17α-methyltestosterone (MT) on sensitivity to female pheromones and courtship behaviors in males of four different cyprinid species: tinfoil barbs (*Barbonymus schwanenfeldii*), redtail sharkminnows (*Epalzeorhynchos bicolor*), goldfish, and zebrafish^92^. MT injections enhanced electroolfactogram measurements to prostaglandins in all four species and elevated courtship behaviors in juvenile redtail sharkminnows. These results suggest a potentially common response system to androgens in related teleost species and set the stage for important comparative insights in follow-up studies.

Moreover, the use of steroid agonists and antagonists have been critical for examining how sex steroids influence aggressive and reproductive behaviors. In the monogamous cichlid *Amatitlania nigrofasciata*, treatment with the non-steroidal AR antagonist flutamide reduces courtship behaviors, but has no effect on aggressive behaviors in males^119^. In male *A. burtoni*, injection of the aromatase inhibitor fadrozole (FAD) reduces the frequency of attacks directed towards males, but does not affect courtship behaviors^120^. Likewise, FAD treatment decreases the rate of attacks directed towards male conspecifics in the weakly electric fish *Gymnotus omarorum*^121^. Therefore, results from *A. burtoni* and weakly electric fish show that estrogen synthesis is required for aggressive, but not courtship behaviors. These findings contrast with those described above for the effects of the synthetic estrogen EE2 on behavior in zebrafish and medaka, which could suggest that EE2 administration affects certain estrogenic signaling pathways differently than naturally synthesized estrogens. Nonetheless, the diversity of teleost fishes in which the influence of estrogenic signaling molecules can be studied, together with an array of available pharmacological approaches, will yield fundamental discoveries on the hormonal mechanisms underlying social behaviors.

In addition to variation in reproductive systems, teleost fishes also exhibit diverse parental care strategies, providing further avenues for exploring the control of mating and aggressive behaviors by steroid hormones across species using pharmacological manipulations. For example, in bluegill (*Lepomis macrochirus*), parental behavior is exhibited solely by males. To test how the neuroendocrine control of mating behavior may be affected by this system, Kindler *et al.* tested the effects of two androgens, 11-KT and T, and cyproterone acetate (CA), a steroidal AR antagonist, on courtship behavior in male bluegills during prespawning and parental periods^122^. Using CA to block AR function may be ideal in teleost species, given the presence of an additional, novel AR paralogous gene. Indeed, since CA is steroidal, it blocks access of androgens to the ligand binding domains of either AR^90^. 11-KT and T implants failed to stimulate nest building in parental males in spring and early summer, while CA treatment reduced reproduction in male bluegill. 11-KT given to nesting males that spawned displayed enhanced courtship behaviors. Treatment with T or CA, however, did not induce these behaviors in nesting bluegill. These results suggest an important role for androgens in controlling courtship behaviors in male bluegill, an effect that may depend on the sensitivity of androgen signaling as a function of spawning or parental care phase. Thus, teleost fishes, like bluegill, may provide a unique opportunity to dissect the role of sex steroids in modulating aggression and mating across distinct life-history stages.

Finally, pharmacological approaches can be used to characterize the control of neural circuitry underlying aggressive and reproductive behaviors via sex steroids. In teleosts, reproductive or territorial state can be expressed via a variety of signals. For example, male Plainfin midshipman (*Porichthys notatus*) perform vocalizations that function for courtship and agonistic purposes^123^. Remage-Healey and Bass asked whether different steroid hormones modulate these vocalizations by combining pharmacological manipulations with electrophysiology. Male midshipman vocalizations are controlled by a hindbrain–spinal circuit, which regulates the frequency and duration of the neuronal firing in the rhythmic vocal motor system. This output determines directly the pair of muscles that modulate the fundamental frequency and duration of vocalizations. Therefore, by recording from neurons in the occipital nerve roots, researchers can measure “fictive” vocalizations in restrained animals in controlled electrophysiology settings, where distinct pharmacological manipulations can be performed. The authors found that androgens, glucocorticoids, and estrogens modulate the duration of vocalizations generated by male midshipman within 15 minutes of administration. These findings suggest that different steroid hormones that are released during social interactions can rapidly alter behaviors that are produced during courtship and agonistic behaviors.

#### 3.2.1. A focus on pharmacological studies in A. burtoni.

In the African cichlid fish *A. burtoni*, the use of different ER, AR, and PR antagonists and agonists, as well as aromatase inhibitors, have led to insights into the role of estrogens, androgens and progestins in the control of aggression and mating (Table 1). The first study to investigate the role of steroids on behavior in *A. burtoni* used the androgen testosterone propionate. Fernald^124^ found that testosterone injection enhanced body coloration and attacks towards males, but did not alter courtship.

Other pharmacology experiments in *A. burtoni* demonstrate the complex regulation of social behavior by steroid hormones. O’Connell and Hofmann^125^ injected DOM or SUB males with different ER, AR, and PR agonists and antagonists and measured aggressive, mating, and submissive behaviors. Male status was confirmed visually before fish were injected with one of the receptor drugs. E_2_ injections increased aggression in DOM and SUB males, while ER antagonism with injections of ICI182780 elevated aggression in SUB males. Surprisingly, after a slight reduction in SUB male aggression following ER antagonism, the rate of aggression paralleled that of the fish given the ER agonist. This pattern was not observed in DOM males, suggesting that the effects depend on social status.

Prior work also suggests roles for androgens and progestins in regulating aggressive and reproductive behaviors. In DOM male *A. burtoni*, 5α-dihydrotestosterone (DHT) administration stimulates whereas CA reduces courtship behavior. However, these manipulations do not alter aggression. In contrast, neither DHT or CA affects the rate of courtship or aggressive behaviors in SUB males. PR manipulations affect distinct behaviors in *A. burtoni* males as well. Injection of dihydroprogesterone increases courtship but not aggression in DOM males, while treatment with the ZK0112993, a PR antagonist, reduces courtship but not aggression. In SUB males, dihydroprogesterone adminstration does not impact aggression or courtship but reduces fleeing. Furthermore, male *A. burtoni* injected with the aromatase inhibitor FAD performed fewer aggressive behaviors compared to uninjected, while courtship behaviors were not impacted^120^. Collectively, these results highlight the complexity of distinct sex steroid signaling systems in controlling social behaviors in *A. burtoni* in a status-specific manner.

The findings from O’Connell and Hofmann led Alward *et al.*^53^ to hypothesize that androgen signaling in particular was critical important for social ascent in male *A. burtoni.* Specifically, because AR receptors alter courtship behavior in DOM, but not SUB males, yet ER manipulation alters aggression in both DOM and SUB males, social opportunity and androgen signaling in conjunction may be important in allowing a rise from SUB to DOM social status. To test this hypothesis, experimentally suppressed SUB males were injected with CA before the opportunity for ascent in an established paradigm. In this assay, a suppressed focal male is housed with a larger suppressor male and 3 females in a central compartment. On either size of this compartment are identical social environments containing males smaller than the focal male. To initiate the opportunity for social ascent, the larger suppressor male is removed during the middle of the night. When the lights come on in the morning, the suppressed male identifies this social opportunity and ascends. This approach has been used frequently and reliably induces social ascent in *A. burtoni* in a controlled experimental setup^53,87,89,126,127^. Males injected with vehicle ascended after removal of the suppressor male, exhibiting increased eye-bar intensity and body coloration and elevated aggression and courtship behaviors. Conversely, males injected with CA showed all the above features except courtship. These results provide further insight into the role of androgens in regulating courtship and that androgen signaling and social opportunity combine to enable social ascent.

### 3.3. Gene editing in teleosts: Emerging comparative models for characterizing the neuroendocrine regulation of social rank

In recent years, gene editing methodologies been used to generate a variety of mutant teleost fishes lacking functional androgenic, estrogenic, or progestogenic receptors, which are particularly useful models for examining the functional significance of sex steroids in controlling social rank. To date, sex steroid signaling systems have been genetically modified in zebrafish, *A. burtoni*, medaka, and tilapia. In this section, we discuss relevant findings from each species and place a special emphasis on *A. burtoni,* as this species has been studied most extensively with respect to the hormonal regulation of social status.

#### 3.3.1. Zebrafish.

Zebrafish lacking functional ARs were made using two gene editing approaches: CRISPR/Cas9 (Clustered Regularly Interspersed Short Palindromic Repeats) and TALENs (Transcription Activator-Like Effector Nucleases)^128^. In this study, courtship behaviors were assessed in wild-type (WT) males, which possess two functional AR alleles, and knockout (KO) males, which possess two non-functional AR alleles. WT males performed more courtship behaviors than KO males, indicating that androgenic signaling is required for courtship in male zebrafish. Moreover, Carver *et al.* investigated the roles of androgen and progesterone signaling in the regulation of aggression by developing AR mutant and PR mutant male zebrafish via CRISPR/Cas9^129^. AR KO fish performed fewer attacks compared to both WT and PR KO fish, whereas PR KO fish performed more attacks compared to both groups. These results suggest that ARs stimulates aggressive behavior, while PR inhibits aggression in male zebrafish. ER KO and aromatase gene knockout zebrafish have also been made^94,96,130,131^, but these mutants have only been used for studies focused on reproductive development and have yet to be used to examine social status traits, such as male-typical aggression or courtship. These mutants have the potential to reveal exciting insights into the complex role played by distinct ERs and aromatase genes in regulating physiological processes and behaviors linked with social rank.

#### 3.3.2. A. burtoni.

Our lab used CRISPR/Cas9 gene editing to generate *A. burtoni* lacking functional ARα, ARβ, or both^27^. We found that DOM ARα mutant males perform fewer mating and territorial behaviors than DOM WT males, but exhibit other DOM-typical traits, including large testes and bright coloration. Conversely, DOM ARβ mutant males display WT-typical levels of mating and territorial behaviors, yet possessed very small testes and drab coloration. Interestingly, both DOM ARα and ARβ mutant males performed WT levels of a physical aggressive behavior called male attacks. Males lacking both receptors (ARαβ mutants) do not perform attacks towards males, however. Taken together, these results suggest social behaviors and reproductive physiology related to social status in *A. burtoni* are regulated by distinct AR genes, suggesting that non-redundant mechanisms control different traits of social status. No ER or aromatase mutant *A. burtoni* have bene engineered, but these fish would provide an excellent opportunity to investigate the role of estrogenic signaling pathways in regulating social status in future work.

#### 3.3.3. Medaka.

ER mutant medaka have been used for studies focused on reproductive development and female mating behavior, but not male-typical aggression or courtship^132–134^. In a recent study, AR mutant medaka were used to determine the role of distinct AR genes (ARα and ARβ) on male-typical reproductive physiology, morphology, and courtship behavior^135^. In terms of results pertaining to the role of sex steroid hormones in the control of social dominance, this study revealed that ARα is necessary for tooth enlargement and the reproductive behavior that stimulates receptivity in females, whereas ARβ is required for fin morphogenesis and sexual motivation in males.

#### 3.3.4. Nile Tilapia.

Nile tilapia lacking functional ERs and aromatase genes have been engineered as well^91,136,137^, but these mutants have not been used to investigate male- or female-typical mating or aggressive behaviors, which would yield insights into the hormonal basis of social dominance. ER, aromatase, and other genetic mutant tilapia may be especially useful for revealing conserved or divergent mechanisms underlying the neuroendocrine control of social status, as this group of teleosts is particularly diverse with espect to reproductive strategies and social structures across species.

## 4. Discussion

Social hierarchies are an essential component of group living for many animals^1,2,6,7^. Although the neuroendocrine control of behaviors associated with dominance (e.g., aggression, mating) have been studied for decades^23,138–142^, we know little about the specific neural and molecular mechanisms through which sex steroids act to regulate social rank, especially in non-traditional animal models. In this review, we highlighted correlational, pharmacological, and molecular genetic studies on the regulation of social rank by sex steroids in teleost fishes. Specifically, we discussed how prior research has provided strong evidence of a relationship between circulating sex steroid levels, their mechanisms of action, and DOM-typical behaviors (e.g., aggression, mating) in males across species of teleost fishes. Furthermore, we highlighted recent studies that have integrated gene editing approaches to study the neuroendocrine regulation of social status in teleosts. Taken together, this research demonstrates the utility of using both pharmacological and state-of-the-art genetic tools to characterize the neuroendocrine regulation of social status both within and among teleost species.

**Figure 1.**
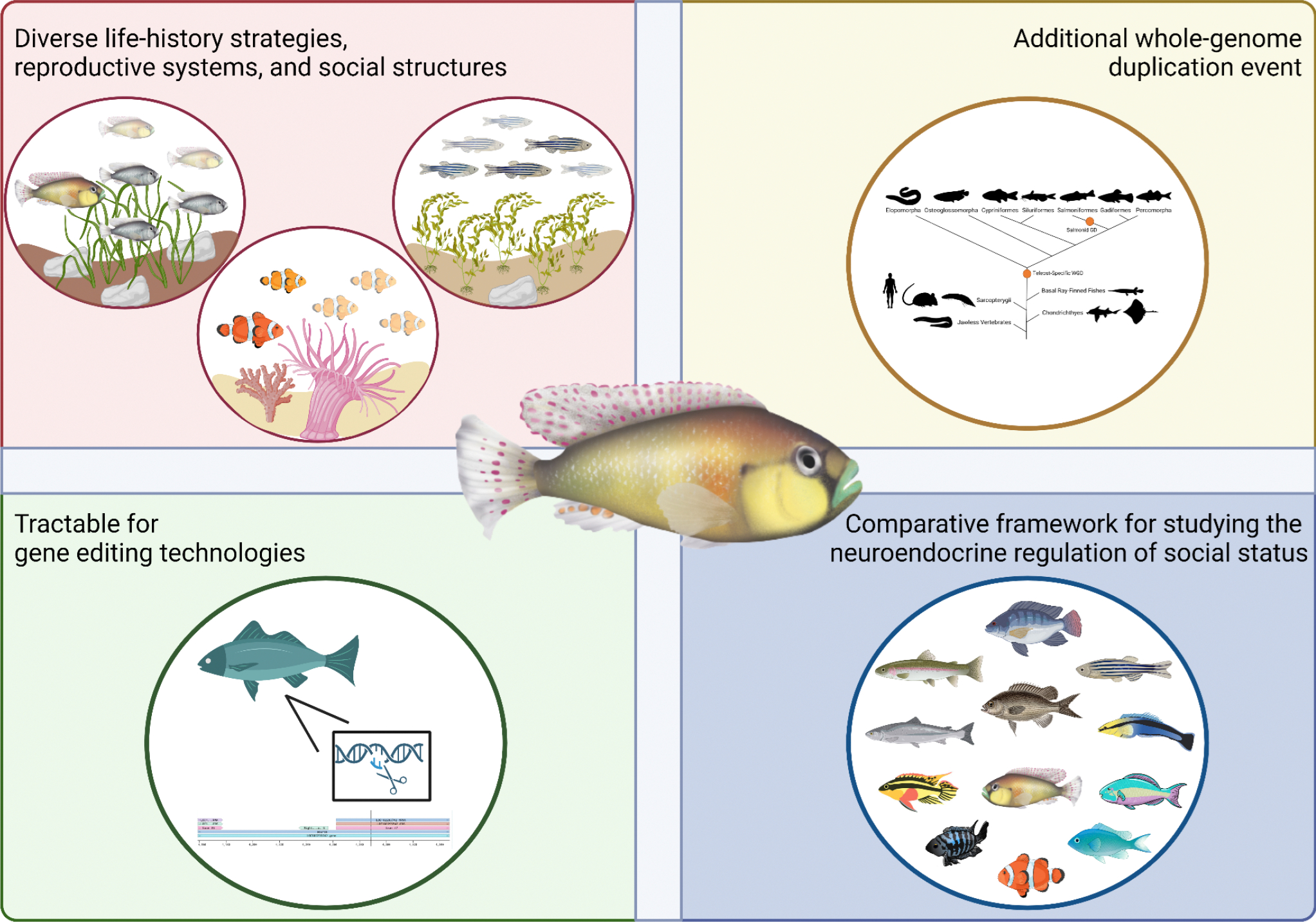
Summary of advantages of using teleost fishes to study the neuroendocrine regulation of social status by sex steroids. **(Top left)** Teleosts exhibit diverse life-history strategies, including variation in reproductive systems and social structures. For example, the African cichlid fish *Astatotilapia burtoni* (left circle) has a polygamous mating system with a male-specific social hierarchy, in which large dominant males (yellow) possess territories that are used to mate with females (gray) and actively defend their territories from small subordinate males (transparent yellow). In contrast, anemonefish (*Amphiprion* and *Premnas* sp.; center circle) have a monogamous mating system with a non-sex-specific social hierarchy, in which a large dominant female (dark orange) mates with a small male (light orange) and actively defends their territory from subordinate, non-breeding individuals (transparent light orange). Zebrafish (*Danio rerio*; right circle) have a polygamous mating system with sex-specific social hierarchies, in which large dominant males and females (blue and white) defend their territories against small subordinate individuals of the same sex (transparent blue and white). **(Top right)** Teleosts possess duplicate gene paralogs due to a teleost-specific whole-genome duplication. **(Center)** The African cichlid fish *A. burtoni* has been an important model organism for characterizing the role of sex steroids in modulating social status. **(Bottom left)** Teleosts are tractable model organisms for using state-of-the-art genetic tools, such as CRISPR/Cas9 gene editing. **(Bottom right)** Teleosts provide an excellent opportunity to study the evolution of the neuroendocrine control of social status by sex steroids using a comparative approach.

Teleost fishes form the largest and most diverse vertebrate clade and, thus, exhibit remarkable variation reproductive strategies, sexual systems, and social structures, among both distant and closely related species (Figure 1)^143–145^. Despite this extraordinary diversity, however, the role of sex steroids in controlling social rank has been studied in surprisingly few teleost species. Teleosts provide an excellent opportunity to elucidate the neural and molecular mechanisms that regulate social rank and to examine how these mechanisms may differ among distant or closely related species that exhibit comparable or distinct life-history strategies. For example, cichlids (family Cichlidae) show remarkable diversity in their reproductive strategies and social structures, even among closely related species. The African cichlid fish *A. burtoni* exhibits a polygamous mating system, in which males exhibit a dominance hierarchy and the female exclusively provides maternal care^87,146^. Conversely, the African cichlid *N. pulcher* has a monogamous mating system, in which all individuals in a population form a dominance hierarchy. In this species, social groups consist of a DOM mating pair and SUB helpers of both sexes, which provide parental care to offspring^147–149^. Some teleosts also exhibit dominance hierarchies within a sexually dynamic system, in which individuals undergo sex change during adulthood in response to changes in their social environment. The most well-studied models of the neural and hormonal control of social rank and its associated behaviors are sex-changing species of reef fish, including bluehead wrasse (*Thalassoma bifasciatum*), anemonefish (*Amphiprion* and *Premnas* sp.), and bluebanded gobies (*Lythrypnus dalli*)^113,150–152^. Future research that uses a comparative framework to characterize the neural and molecular mechanisms controlling social status, especially across species with diverse life-history strategies, will be an invaluable tool for yielding important insights into the evolution of social behavior and its underlying processes in teleost fishes.

Additionally, most studies on sex steroids and social status in teleosts have focused primarily on DOM individuals within social hierarchies. Thus, little is known about the neuroendocrine control of behavior in SUB individuals. While few studies have directly assessed the role of sex steroids in regulating traits associated with subordination, circulating steroids levels (especially androgens) are typically negatively associated with a subordinate social rank^153–155^. There is also considerable evidence that sex steroids interact with other neuromodulators, such as neurotransmitters, to regulate the brain and behavior^156–159^. Further studies are necessary to elucidate the role of between sex steroid hormones in the control of SUB-typical behaviors and to assess whether these mechanisms differ between DOM and SUB individuals. Moreover, prior work that has examined how sex steroids regulate social rank in teleosts have used species in which males form social hierarchies. Thus, the role of sex steroids in regulating social status in female fish, as well as potential sex differences in these underlying mechanisms, are relatively unexplored. These questions can be addressed using teleost species that exhibit female- or non-sex-specific social hierarchies. For example, zebrafish exhibit female-female dominance hierarchies within their social groups^160,161^, whereas in the African cichlid *N. pulcher*, there is a dominance hierarchy among all individuals in a social group, regardless of sex^147,149,162^. Additional research is needed to assess potential sex differences in the regulation of social rank by sex steroids and how these mechanisms may differ among species with different social structures.

Finally, research on the neuroendocrine control of social status in teleosts will benefit by using state-of-the-art genetic methodologies (Figure 1)^20,163,164^. To date, gene editing has only been utilized in a few teleost species, including zebrafish, *A. burtoni*, medaka, and Nile tilapia. The advent of this methodology in teleosts, along with the increased availability and reduced cost of whole-genome sequencing applications in recent years, will make gene editing a feasible approach for studying the neural and molecular mechanisms controlling social status across teleost species. Teleosts also represent a novel opportunity for disentangling the effects of sex steroids on social status and its associated behaviors due to a teleost-specific whole-genome duplication^20,164^. Indeed, there is emerging evidence that some steroid receptors and enzymes within the steroidogenic pathway, including androgen receptors (ARα and ARβ) and aromatase (*cyp19a1* and c*yp19a1a*), have undergone subfunctionalization, in which paralogs of an ancestral gene are maintained after a genome duplication due to the complementary division of functions^41^. For example, teleosts have two androgen receptors, ARα and ARβ, which have distinct roles in regulating traits associated with a DOM social status in male *A. burtoni*. ARα mutants generated via CRISPR/Cas9 gene editing are brightly colored and have large testes, but show deficits in aggressive and mating behaviors, whereas ARβ mutants lack bright coloration and show reduced testes size, but perform normal levels of dominant-typical behaviors^27^. Moreover, because teleosts possess novel duplicate paralogs of ancestral genes, pharmacological manipulations provide little insight into the role of specific steroid receptors and their synthetic enzymes, as this approach often involves the use of non-specific agonists or antagonists that block all subtypes of a steroid receptor or steroidogenic enzyme. Thus, the specificity of gene editing and its more widespread use in teleost fishes will enhance our understanding of how novel steroid receptors and steroidogenic enzymes, such as ARα and ARβ, control social status and to study the evolution of the neuroendocrine regulation of social behavior more broadly.

## 5. Conclusions

Many species are inherently social, and dominance hierarchies are an essential component of establishing and maintaining social stability in group-living animals. While prior research has characterized how sex steroids control aggressive and reproductive behaviors, relatively few studies have examined the neural and molecular mechanisms through which sex steroids regulate social status and its associated behaviors using model organisms that exhibit dominance hierarchies. Teleost fishes are excellent models for addressing these outstanding questions, as they display remarkable variation in their life-history strategies, including reproductive systems and social structures, often among closely related species. Moreover, teleosts possess duplicate paralogs of steroid-related genes due to a teleost-specific whole genome duplication, enabling researchers to disentangle the roles of sex steroids in regulating distinct traits associated with social rank. With the advent of gene editing technologies, such as CRISPR/Cas9, teleost fishes will be invaluable models for elucidating how sex steroids and other neuroendocrine substrates regulate traits linked with social status. More broadly, future research that implements these state-of-the-art tools in teleosts using interdisciplinary and comparative approaches will provide critical insight into how these mechanisms have evolved, and will continue to evolve, in vertebrates.

## Figures and Tables

**Table 1.** Summary of correlational, pharmacological, and gene editing studies in teleost fishes that suggest a role for sex steroids in regulating social status in males.

Scientific Name	Common Name	Family	Hierarchy	Steroid	Circulating Levels	Pharmacology	Gene Editing	References
*Astatotilapia burtoni*	Burton’s mouthbrooder	Cichlidae	♂ only	T	DOM > SUB	T ↑ aggression; DHT ↑ courtship; CA ↓, courtship	ARα mutation ↓, courtship and aggression; ARαβ mutation ↓, aggression	Alward *et al*., 2019^55^, 2020^27^; Fernald, 1976^87^; Maruska and Fernald, 2010^88^; Maruska *et al*, 2013^57^; O’Connell *et al*., 2013^58^; O’Connell and Hofmann, 2012^89^; Parikh *et al*., 2006^59^
				11-KT	DOM > SUB	---	---	Alward *et al*., 2019^55^; Maruska and Fernald, 2010^88^; Maruska *et al,* 2013^57^; Parikh *et al*, 2006^59^
				E_2_	DOM > SUB	E_2_ ↑ aggression; FAD ↓ aggression	N/A	Huffman *et al*, 2013^117^; Maruska and Fernald, 2010^104^; Maruska *et al*, 2013^58^; O’Connell *et al*, 2013^59^; O’Connell and Hofmann, 2012^123^
				PROG	DOM > SUB	DHP ↑ courtship; ZK0112993 ↓, courtship	N/A	O’Connell *et al*, 2013^58^; O’Connell and Hofmann, 2012^89^
*Oreochromis mossambicus*	Mozambique tilapia	Cichlidae	♂ only	11-KT	DOM > SUB	---	---	Golan and Levavi-Sivan, 2013^60^
*Oreochromis niloticus*	Nile tilapia	Cichlidae	♂ only	11-KT	DOM > SUB	---	---	Pfennig *et al*, 2012^61^
*Pundamilia nyererei*	Nyerere’s Victoria cichlid	Cichlidae	♂ only	11-KT	DOM > SUB	---	---	Dijkstra *et al*., 2007^67^
*Cichlasoma dimerus*	Dimerus cichlid	Cichlidae	Sex-specific (♂ only and ♀ only)	T	DOM > SUB	N/A	N/A	Ramallo *et al*, 2015^66^
				11-KT	DOM > SUB	---	---	Morandini *et al*, 2014^65^; Ramallo *et al*, 2015^66^
				E_2_	DOM < SUB	N/A	N/A	Ramallo *et al*., 2015^66^
*Neolamprologus pulcher*	Daffodil cichlid	Cichlidae	All individuals (♂ and ♀)	T	DOM > SUB	N/A	N/A	Aubin-Horth *et al*., 2007^62^; Desjardins *et al*., 2008^63^
				11-KT	DOM > SUB	---	---	Desjardins *et al*, 2008^63^; Taves *et al*, 2009^64^
*Oncorhynchus mykiss*	Rainbow trout	Salmonidae	♂ only	T	DOM > SUB	N/A	N/A	Cardwell *et al*., 1996^68^; Liley and Kroon, 1995^69^
				11-KT	DOM > SUB	---	---	Liley and Kroon, 1995^69^
*Salmo trutta*	Brown trout	Salmonidae	♂ only	T	DOM = SUB	N/A	N/A	Cardwell *et al*, 1996^68^
				11-KT	DOM > SUB	---	---	Cardwell *et al*, 1996^68^
*Salvelinus alpinus*	Arctic char	Salmonidae	♂ only	T	DOM > SUB	N/A	N/A	Elofsson *et al*., 2000^70^
				11-KT	DOM > SUB	---	---	Elofsson *et al*., 2000^70^
*Sparisoma viride*	Stoplight parrotfish	Scaridae	♂ only	T	DOM > SUB	N/A	N/A	Cardwell and Liley, 1991^71^
				11-KT	DOM > SUB	---	---	Cardwell and Liley, 1991^71^
*Chromis dispilus*	New Zealand demoiselle	Pomacentridae	♂ only	T	DOM > SUB	N/A	N/A	Pankhurst and Barnett, 1993^72^
				11-KT	DOM > SUB	---	---	Pankhurstand Barnett, 1993^72^
*Symphodus ocellatus*	Ocellated wrasse	Labridae	♂ only	T	DOM = SUB	N/A	N/A	Stiver *et al*., 2015^73^
				11-KT	DOM > SUB	---	---	Stiver *et al*., 2015^73^
				E_2_	DOM = SUB	N/A	N/A	Stiver *et al*., 2015^73^
*Oryzias latipes*	Japanese medaka	Adrianichthyidae	♂ only	T	N/A	N/A	ARα mutation ↓, reproductive behavior; ARαβ mutation ↓, sexual motivation	Ogino *et al*., 2023^91^
				11-KT	DOM > SUB	---	---	Kagawa *et al*., 2017^74^
				E_2_	N/A	E_2_ and EE2 ↓ courtship	N/A	Balch *et al*, 2004^92^; Oshima *et al*, 2003^93^
*Danio rerio*	Zebrafish	Cyprinidae	Sex-specific (♂ only and ♀ only)	T	N/A	BPA ↓ courtship, ↑ aggression	ARαβ KO ↓ courtship and aggression	Carver *et al*., 2021^94^; Lu *et al*., 2017^95^; Yong *et al*., 2017^96^
				11-KT	DOM > SUB	---	---	Filby *et al*., 2010^75^
				E_2_		EE2 ↓ aggression and ↑ social preference	N/A	Fenske *et al*., 2020^97^
				PROG	N/A	N/A	PR KO ↑ aggression	Carver *et al*., 2021^94^

Results of studies that measured circulating sex steroid levels or the effects of pharmacological manipulations or genetic deletion of sex steroid receptors on dominant-typical behaviors In teleosts. Findings that are reported In this table for pharmacological studies are from dominant males. Only studies that Investigated these mechanisms In teleost species that form social hierarchies are shown (see Section 3 for additional studies that examined the role of sex steroids In regulating aggressive or mating behaviors In males of species that do not form social hierarchies).

Abbreviations: 11-KT, 11-ketotestosterone; ARα, androgen receptor alpha; ARβ, androgen receptor beta; BPA, bisphenol A; CA, cyproterone acetate; DHT, 5α-dihydrotestosterone; DHP, 5α-dihydroprogesterone; DOM, dominant; E_2_, 17β-estradiol; EE2, ethinyl estradiol; FAD, fadrozole; KO, knockout; MT, 17α-methyltestosterone; PROG, progesterone; SUB, subordinate; T, testosterone.

## Data Availability

No data are associated with this article.
